# Substrate Stiffness of Bone Microenvironment Controls Functions of Pre-Osteoblasts and Fibroblasts In Vitro

**DOI:** 10.3390/biomimetics8040344

**Published:** 2023-08-04

**Authors:** Shenghan Gao, Bo Chen, Min Gao, Yue Xu, Xueyi Yang, Chun Yang, Shaoxia Pan

**Affiliations:** 1 Department of Prosthodontics, Peking University School and Hospital of Stomatology & National Center for Stomatology & National Clinical Research Center for Oral Diseases & National Engineering Research Center of Oral Biomaterials and Digital Medical Devices, Beijing Key Laboratory of Digital Stomatology, Research Center of Engineering and Technology for Computerized Dentistry Ministry of Health, NMPA Key Laboratory for Dental Materials, Central Laboratory, Peking University School and Hospital of Stomatology, No. 22, Zhongguancun South Avenue, Haidian District, Beijing 100081, China; 2 Department of Implantology, Peking University School and Hospital of Stomatology & National Center for Stomatology & National Clinical Research Center for Oral Diseases & National Engineering Research Center of Oral Biomaterials and Digital Medical Devices, No. 22, Zhongguancun South Avenue, Haidian District, Beijing 100081, China; 3 Department of Geriatric Dentistry, Peking University School and Hospital of Stomatology & National Center for Stomatology & National Clinical Research Center for Oral Diseases & National Engineering Research Center of Oral Biomaterials and Digital Medical Devices, No. 22, Zhongguancun South Avenue, Haidian District, Beijing 100081, China; 4 Institute of Biomechanics and Medical Engineering, School of Aerospace Engineering, Tsinghua University, Beijing 100084, China

**Keywords:** substrate stiffness, pre-osteoblast, fibroblast, bone regeneration, biomimetic microenvironment

## Abstract

The formation of bone in a bone defect is accomplished by osteoblasts, while the over activation of fibroblasts promotes fibrosis. However, it is not clear how the extracellular matrix stiffness of the bone-regeneration microenvironment affects the function of osteoblasts and fibroblasts. This study aim to investigate the effect of bone-regeneration microenvironment stiffness on cell adhesion, cell proliferation, cell differentiation, synthesizing matrix ability and its potential mechanisms in mechanotransduction, in pre-osteoblasts and fibroblasts. Polyacrylamide substrates mimicking the matrix stiffness of different stages of the bone-healing process (15 kPa, mimic granulation tissue; 35 kPa, mimic osteoid; 150 kPa, mimic calcified bone matrix) were prepared. Mouse pre-osteoblasts MC3T3-E1 and mouse fibroblasts NIH3T3 were plated on three types of substrates, respectively. There were significant differences in the adhesion of pre-osteoblasts and fibroblasts on different polyacrylamide substrates. Runx2 expression increased with increasing substrate stiffness in pre-osteoblasts, while no statistical differences were found in the Acta2 expression in fibroblasts on three substrates. OPN expression in pre-osteoblasts, as well as Fn1 and Col1a1 expression in fibroblasts, decreased with increasing stiffness. The difference between the cell traction force generated by pre-osteoblasts and fibroblasts on substrates was also found. Our results indicated that substrate stiffness is a potent regulator of pre-osteoblasts and fibroblasts with the ability of promoting osteogenic differentiation of pre-osteoblasts, while having no effect on myofibroblast differentiation of fibroblasts.

## 1. Introduction

The formation of bone in a bone defect is accomplished by osteoblasts [1], while the over-activation of fibroblasts promotes fibrosis [2]. Previous studies have typically used barrier membranes to isolate fibroblasts from the bone defect space and avoid connective tissue occupying the empty defect space [3]. However, the fact that fibroblasts are also present and play a crucial role in the bone-regeneration microenvironment was ignored [2,4].

The inflammatory phase and repair phase are both important in the bone-regeneration process [2]. In the early stages of inflammation, fibroblasts, osteoblasts, mesenchymal stem cells (MSCs) and endothelial cells are recruited [5,6]. Endothelial cells are recruited to participate in new blood vessel formation. The adhesion, migration and proliferation of endothelial cells were enhanced on a substrate with high stiffness [7]. However, osteoblasts and fibroblasts work together to promote bone regeneration, while fibroblasts also compete against osteoblasts to occupy the bone defect site [8]. Therefore, though many cells are recruited, we focus on osteoblasts and fibroblasts in this study. After new blood vessels invade, the hematoma at the bone defect site transforms into granulation tissue, and bone gradually enhances the repair processes [9]. The cellular components of granulation tissue that contribute to bone regeneration include fibroblasts, osteoprogenitor cells and MSCs [9].

**Figure 1:**
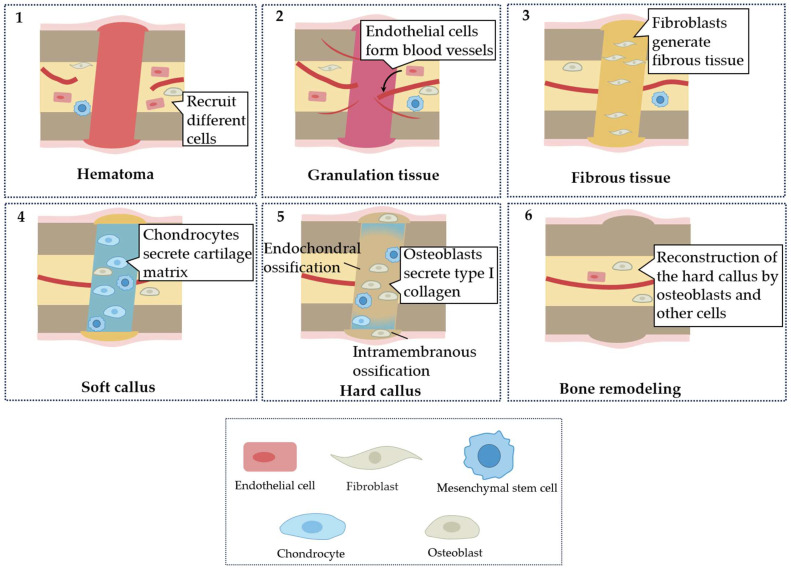
Process of bone regeneration. The phases of bone regeneration can be divided into (**1**) hematoma, (**2**) granulation tissue, (**3**) fibrous tissue, (**4**) soft callus, (**5**) hard callus and (**6**) bone remodeling. In each phase, different cells play the dominant roles.

Fibroblasts and osteoblasts secret matrix to replace granulation tissue and play a crucial part in achieving bone regeneration. In the repair phase, fibroblasts are recruited and activated for a fibrotic response to generate a transient collagenous matrix with enhanced mechanical strength, to replace the granulation tissue [9]. Failure to terminate the fibrotic response in pathological conditions, such as polytrauma of bone and skeletal muscle, induces pathological scarring, termed fibrosis, leading to excess collagen deposition [10,11]. The accumulation of fibrous tissue within the fracture callus interferes with bone defect consolidation [11]. After the fibrotic response, endochondral ossification occurs in the inter-cortical and cancellous bone areas, while intramembranous ossification occurs in the subperiosteal area and the adjacent soft tissue areas [12]. In the inter-cortical and cancellous bone areas, MSCs proliferate and further differentiate into chondrocytes, which secrete cartilage matrix that transforms the fiber-rich granulation tissue into a soft callus with higher stiffness [9]. Then, osteoblasts secrete type I collagen and participate in bone mineralization, transforming soft callus into hard callus [13]. In the subperiosteal area and the adjacent soft tissue areas, pre-osteoblasts and MSCs are recruited. These cells differentiate into osteoblasts, which secrete extracellular matrix proteins and generate bone mineral crystals, and directly form the hard callus under the periosteum [9,14]. Then, the new bone is remodeled by osteoblasts, osteoclasts and other cells, to restore the initial structure of bone [9]. In addition, scaffolds were manufactured to promote bone regeneration by regulating cell behavior. For example, polyether ether ketone (PEEK)/titanium dioxide (TiO_2_) scaffold coating hydroxyapatite (HA) demonstrated a strong potential to support new bone regeneration via promoting pre-osteoblast adhesion, proliferation and osteoblastic differentiation [15]. Figure 1 illustrates the different stages of bone regeneration and the role of different cells. Altogether, the bone matrix gradually becomes stiffer during the process of bone regeneration.

The extracellular matrix (ECM) is a scaffold around the cell that supports and provides biochemical signals and biophysical cues to the cell [16]. ECM stiffness, one of the biophysical cues, has been shown to regulate many cellular behaviors, including proliferation, morphology and differentiation [17,18]. Therefore, studies on the ECM have especially focused on matrix stiffness [19]. It has been found that the functions of both osteoblasts and fibroblasts are regulated by ECM stiffness [20,21]. Fibroblasts and osteoblasts play a role in the early and late stages of the bone-repair phase, respectively, in which the bone ECM undergoes a process of change from soft matrix to stiff matrix [9]. Thus, changes in ECM stiffness may affect the function of fibroblasts and osteoblasts. However, different types of ECM protein were coated on substrates with various stiffnesses in previous studies [20,21]. ECM protein ligands combine with adhesion receptors of cells and play a crucial role in determining cellular response [22]. Integrins are adhesion receptors that engage extracellular matrix ligands and coordinate the assembly of the cytoskeleton [23]. Integrins binding to extracellular matrix ligands regulate cell traction forces and lead to downstream activation of signaling pathways, which affects the cellular response [24]. Therefore, the effect of ECM stiffness change in the bone-regeneration microenvironment on osteoblast and fibroblast function remains unclear.

In this study, three types of polyacrylamide (PAAm) substrate were employed to mimic the stiffness of the bone-regeneration microenvironment at different stages. Type I collagen was coated on the surface of the PAAm substrate. Then, mouse pre-osteoblast MC3T3-E1 and mouse fibroblast NIH3T3 were plated on the PAAm substrate. Cells use integrin, a mechanoreceptor, to detect ECM stiffness [25]. Integrin then regulates the cell response via activating downstream signals [26]. Cells with various integrin expression levels exhibited different responses to ECM stiffness [27]. Thus, we examine the integrin expression levels of pre-osteoblasts and fibroblasts. In addition, the effect of different PAAm substrates on cell adhesion, cell proliferation and differentiation were assessed, respectively. The cell contraction force (CTF) of MC3T3 and NIH3T3 on different substrates were analyzed.

## 2. Materials and Methods

**Cell culture.** MC3T3-E1 osteoblastic cells and NIH3T3 fibroblast cells were maintained in Dulbecco’s Modified Eagle Medium with 10% fetal bovine serum and 1% penicillin–streptomycin at 37 °C in 5% CO_2_. The medium was changed every other day. Every 3 days, cells were sub-cultured (1:3) using 0.25% trypsin with 1 mM EDTA. MC3T3-E1 and NIH3T3 were plated onto the substrate at the density of 2 × 10^4^/mL for immunostaining, cell proliferation and traction force microscopy, and the density of 8 × 10^4^/mL for real-time PCR analysis.

**Polyacrylamide** (**PAAm**) **substrate preparation.** We prepared polyacrylamide gels with variable Young’s moduli using different proportions of acrylamide and bis-acrylamide, as shown in Table 1. The protocol was following the method introduced by Plotnikov et al. [28]. Briefly, acrylamide and bis-acrylamide with specific concentrations were mixed and the mixture was allowed to polymerize into gel on the glass slide. Then, the PAAm substrate was covered by sulfosuccinimidyl-6-[4′-azido-2′-nitrophenylamino] hexanoate (Sulfo-SANPAH; Pierce, Rockford, IL, USA) and exposed to UV light for 15 min. Then, the substrate was washed three times using Phosphate-Buffered Saline (PBS). Type I collagen at 0.1 mg/mL was incubated overnight at 4 °C with the substrate and crosslinked to the substrate surface for cell adhesion using the Sulfo-SANPAH reagent [29]. Before plating the cells, the substrate was washed three times using PBS to remove unbinding type I collagen.

**Characterization of various elastic PAAm substrates.** Young’s moduli of three substrates were measured by the universal testing machine (TA, Newcastle, DE, USA). A 3 mm diameter metal spherical probe was used to press into the PAAm substrate. The stress on the probe and the press distance of the probe were collected. The press distance of the probe is considered to be the strain of the substrate. Young’s moduli was evaluated by the following equation:

(1) $$F=\left(\frac{4}{3}\right)E\times \frac{\frac{R1}{2d3}}{2}$$

(2) $$\frac{1}{E^{*}}=\frac{1-\nu_{1}^{2}}{E_{1}}+\frac{1-\nu_{2}^{2}}{E_{2}}$$

*F*—stress, E*—plane strain Young’s Modulus of the substrate and probe, *R*—the diameter of the probe, *d*—the strain of the substrate, *E*_1_—Young’s moduli of the substrate, *E*_2_—Young’s moduli of the probe, $\nu_{1}$—Poisson’s ratio of the substrate, $\nu_{2}$—Poisson’s ratio of the probe.

$\nu_{1}$, Poisson’s ratio of the PAAm substrate, is approximately 0.5 [30]. Young’s modulus of the metal probe, is much greater than Young’s modulus of the substrate. Therefore, the metal probe is considered to be a rigid body without deformation and only the plane strain Young’s modulus of the substrate should be considered in Equation (2).

**Immunostaining.** After 1 day of incubation, the cells were fixed in 4% paraformaldehyde for 30 min, and permeabilized by 0.1% Triton X-100 for 5 min. The cells were blocked in 5% goat serum for 1 h at room temperature and then incubated with primary antibody of vinculin (1:200, proteintech, Rosemont, IL, USA) overnight at 4 °C and FITC-conjugated secondary antibody (1:400, earthox, Hillsdale, WY, USA) for 2 h at room temperature. For cytoskeleton staining, the cells were stained with phalloidin. The images were captured by a laser scanning confocal microscope (Nikon, Tokyo, Japan).

**Cell proliferation.** After incubating for 1, 3, 5 and 7 days, cell proliferation was investigated using a Cell Counting Kit-8 (CCK-8, solarbio, Beijing, China) according to the manufacturer’s instructions. Briefly, the cells were incubated with 10% CCK-8 solution for 1 h at 37 °C, and the spectrophotometric absorbance of solution was measured using a microplate reader (TECAN, Switzerland) at 450 nm.

**Quantitative PCR** (**qPCR**) **analysis.** After incubating for 7 days, total RNA of the cells was extracted using TRIzol reagent (Invitrogen, Carlsbad, USA). The concentration and purity of mRNA were measured using a microplate spectrophotometer. A RevertAid First Strand cDNA Synthesis Kit (Thermo Scientific, Waltham, MA, USA) was employed to remove gDNA and synthesize cDNA according to the manufacturer’s instructions. Quantitative PCR analysis was conducted using SYBR Green Realtime PCR Master Mix (TOYOBA, Osaka, Japan). Gene expression data were quantified relative to GAPDH, calculated by the comparative Ct method (2^−(ΔΔC(t))^) and normalized to data on soft substrate. The primer sequences of genes are in Table 2.

**Traction force microscopy.** The protocol for traction force microscopy was previously introduced [28]. Briefly, 100 nm green fluorescent microspheres were linked to the surface of 39 μm thick polyacrylamide (PAAm) substrates to visualize for cell-induced substrate deformation. Then, type I collagen at 0.1 mg/mL was cross-linked to the substrate surface using Sulfo-SANPAH, as previously introduced. The cells were plated on the substrate for 2 days before measuring the cell traction force (CTF). Images of the microspheres and cells on the substrates were firstly captured by a laser scanning confocal microscope (Nikon, Tokyo, Japan). For measuring the CTF, 10% SDS was perfused into a confocal dish to detach the cells from the substrate, and immediately captured images of microspheres on the undeform substrates. We analyzed the microsphere displacement and the CTF using custom-generated Matlab programs. Correlation-based particle tracking velocimetry (PTV) was used to quantify the displacement field of the microspheres on the substrates, which corresponds to the cell-induced deformation of the substrate. We interpolated the displacement field onto a regularized grid and calculated the stress field by regularized-Fourier transform traction cytometry [28]. The regularization parameter was set as 2 × 10^−6^ in order to achieve high resolution for the traction maps.

**Statistical Analysis.** The data were analyzed with a statistical software program (IBM SPSS Statistics, v20.0; IBM Corp, Armonk, NY, USA). One-way analysis of variance (ANOVA) and post hoc test (LSD) were used to compare differences among experimental groups. Data were considered statistically significant if the *p* value was less than 0.05.

## 3. Result

### 3.1. Fabrication of Polyacrylamide Substrates with Varied Stiffness

**Figure 2:**
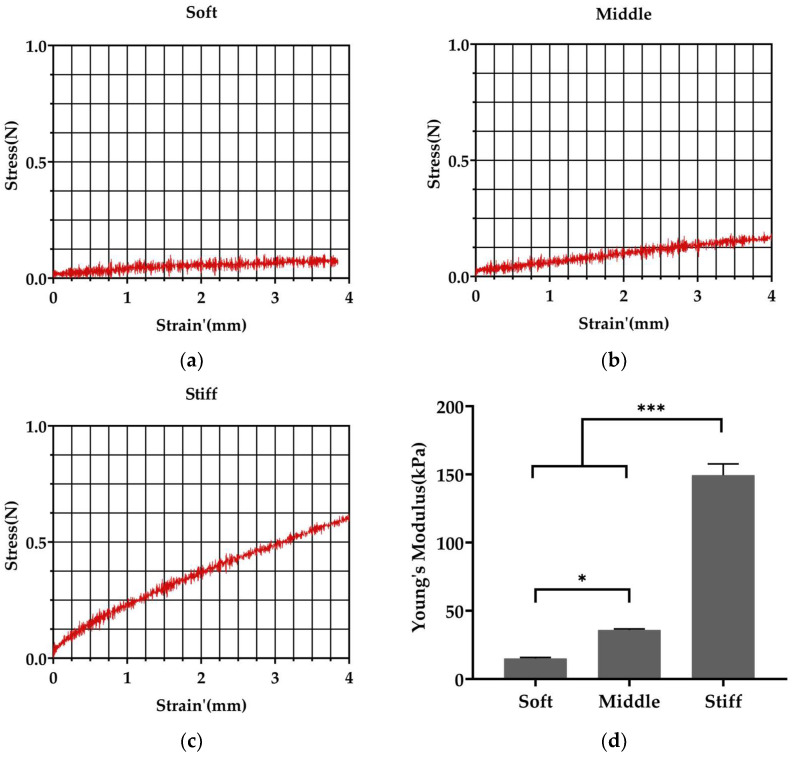
Fabricated polyacrylamide substrates with varied stiffness. (**a**–**c**) Stress–strain curves of soft, middle and stiff substrates prepared with different ratios of acrylamide and bis-acrylamide. Strain’ = Strain^3/2^. (**d**) Soft, middle and stiff substrates displayed stiffnesses of 15, 35 and 150 kPa, respectively. Three independent experiments (n = 3). Data are presented as mean ± standard deviation. * *p* < 0.05, *** *p* < 0.001.

Fabrication of PAAm substrates was achieved through mixing different agent proportions as Table 1. A 3 mm diameter metal spherical probe was used to test the stiffness of the PAAm substrate. The pressed depth of probe in the PAAm substrate was recorded as strain. Strain’ was equal to Strain^3/2^ for the calculation of Young’s moduli. In our study, the mechanical test showed an increase in stiffness with an increasing proportion of acrylamide and bis-acrylamide. The stiffness of soft, middle and stiff substrates was approximately 15, 35, and 150 kPa (Figure 2d), respectively, mimicking the stiffness of granulation tissue [31], osteoids [32] and bone matrix, when starting to calcify [32].

### 3.2. Pre-Osteoblasts Exhibit Higher Level of mRNA Expression of the Integrin Subunits Binding to Type I Collagen Than Fibroblasts

**Figure 3:**
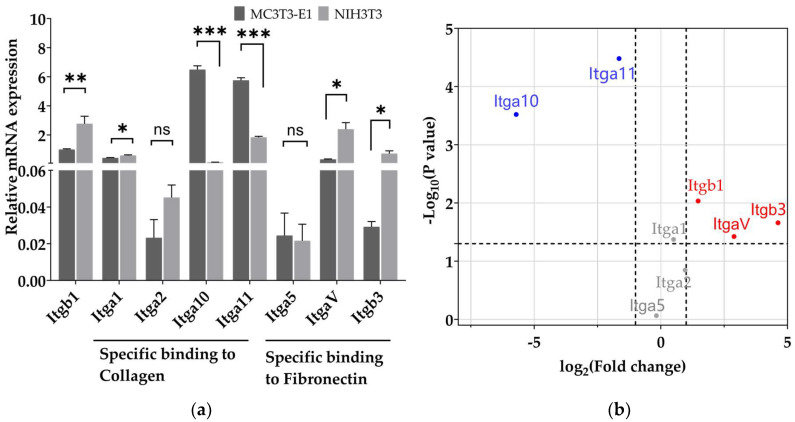
The mRNA expression levels of integrins in pre-osteoblasts and fibroblasts. (**a**) The mRNA expression level of integrin subunits that specifically bind type I collagen and fibronectin in pre-osteoblasts and fibroblasts. Data are presented as mean ± standard error of the mean, * *p* < 0.05, ** *p* < 0.01, *** *p* < 0.001. ns refers to no statistically significant difference. (**b**) Differences in integrin subunit mRNA expression levels in fibroblasts compared to pre-osteoblasts. Itga10 and Itga11 (blue) are at a lower expression level in fibroblasts while Itgb1, Itgb3, and ItgaV (red) are at a higher expression level in fibroblasts compared to pre-osteoblast. There was no statistically significant difference of Itga1, Itga2 and Itga5 (grey) expression level in pre-osteoblasts and fibroblasts. Three independent experiments (n = 3).

The PAAm substrate surface needs to cross-link protein for cell adhesion. Type I collagen and fibronectin are proteins generally coated on the surface of substrates for cell adhesion [22]. Collagen is the most abundant component of the organic ECM in bones and type I collagen accounts for 90% of the total collagen [33]. In order to mimic the protein component of the bone-regenerative microenvironment and promote pre-osteoblast adhesion, type I collagen was coated on the substrate surface. Integrin is essential for cell adhesion as integrin can bind to extracellular ligands [34]. The gene expression level of integrin which binds to type I collagen and fibronectin (Figure 3a) was examined. Figure 3b illustrates an overview of the expression differences as well as the statistical significance. Itga10 and Itga11, which are α-subunit members of integrin binding to type I collagen [23], were more highly expressed in pre-osteoblasts compared to fibroblasts. ItgaV and Itgb3, which are subunits binding to fibronectin, had lower expression in pre-osteoblasts compared to fibroblasts. Itgb1, which is a subunit that enables binding to both type I collagen and fibronectin [23], had lower expression in pre-osteoblasts compared to fibroblasts. Additionally, there is no statistical significance in the mRNA level of Itga1, Itga2 and Itga5 between pre-osteoblasts and fibroblasts.

### 3.3. Stiffer Substrate Increases the Length of Focal Adhesion in Pre-Osteoblasts and Reduces the Length of Focal Adhesion in Fibroblasts

**Figure 4:**
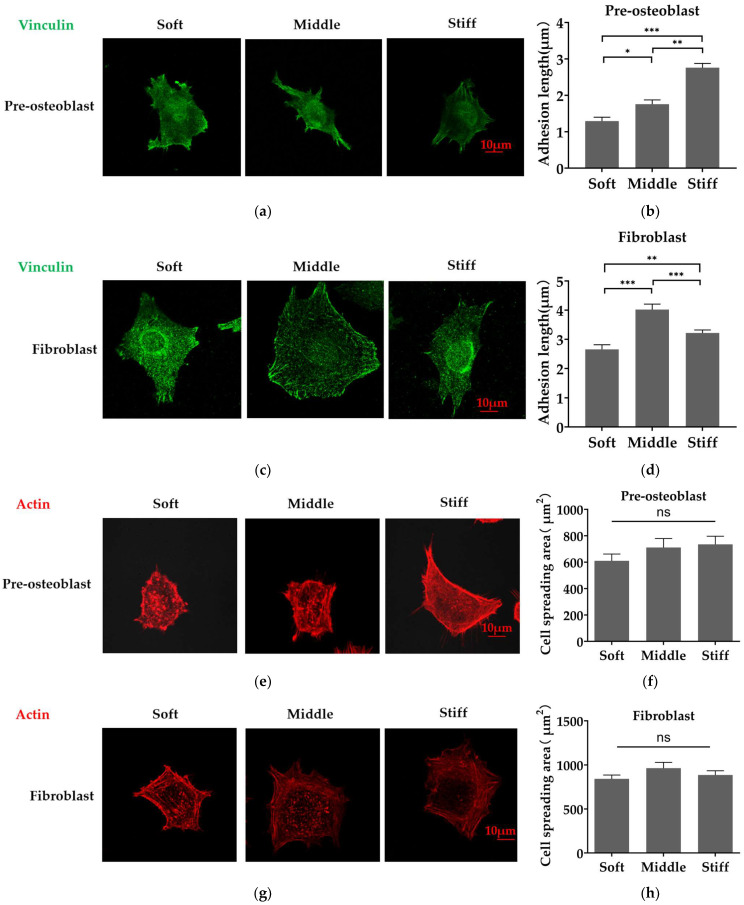
Substrate stiffness regulates the distribution of Vinculin and the assembly of actin fibers in pre-osteoblasts and fibroblasts. (**a**,**c**) Immunofluorescence analysis of focal adhesion in (**a**) pre-osteoblasts and (**c**) fibroblasts using FITC-Vinculin (green). (**b**,**d**) Quantitative analysis of focal adhesion length of (**b**) pre-osteoblasts and (**d**) fibroblasts (at least 3 focal adhesions were measured for each cell in each condition, 3 independent experiments). (**e**,**g**) Immunofluorescence analysis of the cytoskeleton of (**e**) pre-osteoblasts and (**g**) fibroblasts staining by phalloidin (red). (**f**,**h**) Quantification of the corresponding cell spreading area of (**f**) pre-osteoblasts and (**h**) fibroblasts. Three independent experiments (n = 3). Scale bar, 10 μm. Data are presented as mean ± standard error of the mean, * *p* < 0.05, ** *p* < 0.01, *** *p* < 0.001, ns refers to no statistically significant difference.

To examine the effect of stiffness on cell adhesion, pre-osteoblasts and fibroblasts were plated on soft, middle and stiff substrates. The assembly of vinculin and actin stress fibers and cell spreading area were assessed after 1 day of incubation. Fluorescent images revealed that pre-osteoblasts plated on stiff substrate formed longer focal adhesions and a higher organized F-actin network (Figure 4a,e). Statistical data revealed that the focal adhesion in pre-osteoblasts was significantly longer on the middle substrate and stiff substrate (Figure 4b,f). However, fibroblasts cultured on the middle substrate formed longer focal adhesions and form an organized F-actin network on three substrates (Figure 4c,g). Statistical data reveal that the focal adhesion in fibroblasts was significantly longer and peaked on the middle substrate (Figure 4d). In addition, there was no statistically significant difference in the cell spreading area of the two cell types on three substrates (Figure 4h).

### 3.4. Proliferation of Fibroblasts Is Faster Than Pre-Osteoblasts on Three Substrates

**Figure 5:**
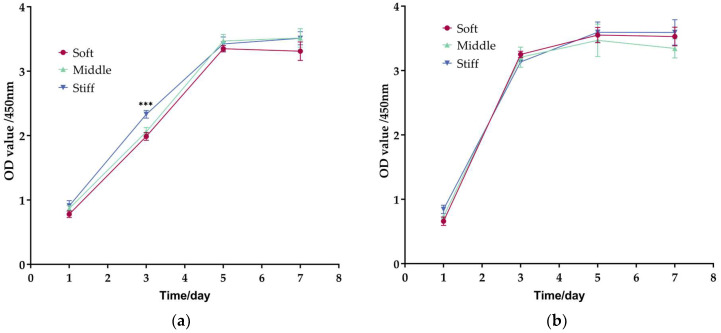
Cell proliferation assay in pre-osteoblasts and fibroblasts. Cell proliferation of (**a**) Pre-osteoblasts and (**b**) fibroblasts on soft, middle and stiff substrates. Three independent experiments (n = 3). Data are presented as mean ± standard error of the mean, *** *p* < 0.001 compared to soft substrate.

The cell proliferation assay revealed a similar proliferation potential of two cell types on all substrates on Day 5 and Day 7 (Figure 5). However, pre-osteoblasts exhibited a higher proliferation rate on stiff substrate on Day 3 compared with soft and middle substrate (Figure 5a). In addition, the cell proliferation rate of fibroblasts was faster than pre-osteoblasts at Day 3 (Figure 5a,b).

### 3.5. Substrate Stiffness Modulates Cell Differentiation and Matrix Secretion of Pre-Osteoblasts and Fibroblasts

**Figure 6:**
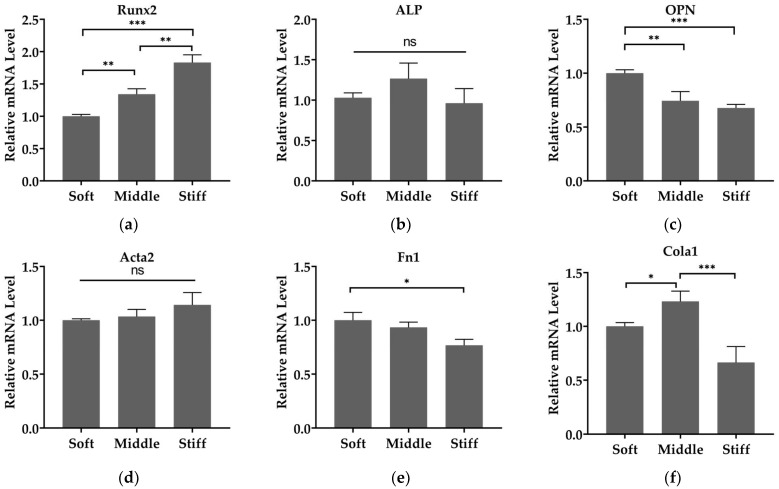
Substrate stiffness modulates cell differentiation and matrix secretion of pre-osteoblasts and fibroblasts. The mRNA level of (**a**) Runx2, (**b**) ALP and (**c**) OPN in pre-osteoblasts and (**d**) Acta2, (**e**) Fn1 and (**f**) Cola1 in fibroblasts. Gene expressions are relative to that of GAPDH and normalized to those on soft substrate. Three independent experiments (n = 3). Data are presented as mean ± standard error of the mean, * *p* < 0.05, ** *p* < 0.01, *** *p* < 0.001, ns refers to no statistically significant difference.

We then investigated the effect of substrate stiffness on cell differentiation and matrix secretion. The mRNA expressions of Runx2 and OPN in pre-osteoblasts and of Acta2, Fn1 and Cola1 in fibroblasts were assessed. Expression of Runx2, the prime control gene of osteoblastogenesis [20], was increased as substrate stiffness increased (Figure 6a). However, no statistical differences were found in the expression of ALP, a specific marker for cells in the osteoblast lineage [35], on three substrates (Figure 6b), and this phenomenon was also present in the expression of Acta2, a marker characterizing fibroblast differentiation into myofibroblasts [36] (Figure 6d). The mRNAs of matrix proteins OPN, a marker associated with mature osteoblasts [35], decreased as the substrate stiffness increased (Figure 6c). Similarly, the expression levels of Fn1 and Cola1 were significantly reduced on stiff substrate compared to soft and middle substrates (Figure 6e,f).

### 3.6. Stiffer Substrate Enhances Cell Traction Force of Pre-Osteoblast While Reducing Cell Traction Force of Fibroblast

**Figure 7:**
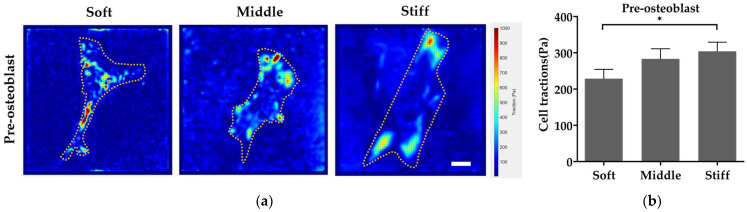

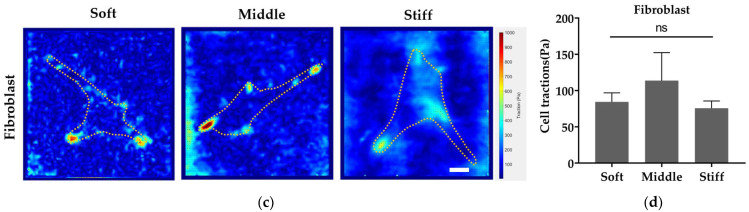
Stiff substrate increases cell traction force of pre-osteoblasts and decreases cell traction force of fibroblasts. Effect of substrate stiffness on cell traction force (color coding) of (**a**) pre-osteoblasts and (**c**) fibroblasts. Quantitative statistics of cell traction force of (**b**) pre-osteoblasts and (**d**) fibroblasts. Three independent experiments (n = 3). Data are presented as mean ± standard error of the mean. * *p* < 0.05, ns refers to no statistically significant difference. Scale bar, 20 µm.

To probe the mechanism of substrate stiffness modulating cell behaviors, the cell traction force (CTF), an important component in mechanotransduction, was detected by traction force microscopy. From traction maps, pre-osteoblasts generated a stronger CTF on stiff substrate (Figure 7a,b). The CTF of fibroblasts was firstly increased with the increase in stiffness from soft to middle substrate (Figure 7c,d). However, the CTF of fibroblasts reduced with the increase in stiffness from middle to stiff substrate, although there was no statistical difference between the three substrates (Figure 7c,d). In addition, a larger magnitude of CTF was found in pre-osteoblasts compared to fibroblasts (Figure 7b,d).

## 4. Discussion

Tissue engineering with mimicking of the tissue microenvironment for tissue regeneration, via regulating endogenous cells, has been introduced [37]. In the bone-regeneration phase, bone ECM gradually changes from soft matrix to rigid matrix [9]. Different types of ECM are generated upon the bone regeneration and the mechanical properties of newly forming bone ECM change quite dynamically. In the early stage, hematoma provides a soft ECM that enables cells to adhere, produce an amount of extracellular matrix protein and remodel the ECM structure into granulation tissue [2]. Then, chondrocytes secrete a semi-rigid cartilage matrix, transforming granulation tissue cartilage-rich soft callus [9]. Subsequently, the soft callus is removed, and hard callus, which contains mineralized extracellular matrix tissue and is proteinaceous, is formed by osteoblasts [13]. Finally, hard callus is remodeled towards the cortical or trabecular bone [9]. The bone ECM stiffens during this whole process [2]. In previous studies, different proteins were coated on the substrate surface [20,38]. The effects of surface features, protein and substrate stiffness resulted in a complex process [35], interfering with the intercomparison of results. The proteins coated on substrates in previous studies were different [20,21]. Thus, the effect of stiffness presenting in the bone-regeneration process on fibroblasts and pre-osteoblasts remains unclear. 

In our study, we prepared PAAm substrates with different stiffness, and coated type I collagen on the surface of the substrate to mimic the bone-regeneration microenvironment at different stages. We choose PAAm to fabricate the substrate because the elasticity of PAAm substrate can be tuned to mimic the stiffness of many biological tissues [39]. For example, soft substrate (0.1–1 kPa) was used to mimic brain, middle substrate (8–17 kPa) was used to mimic muscle, and comparatively stiff substrate (25–40 kPa) was used to mimic collagenous bone [32]. In our study, 15 kPa, 35 kPa and 150 kPa PAAm substrates were prepared to mimic ECM with different stiffness magnitude presented in the bone-regeneration process (Figure 2). Soft substrate (15 kPa) was prepared to mimic the stiffness of granulation tissue [31]. Middle substrate (35 kPa) was prepared to mimic the stiffness of osteoid, which is secreted by osteoblasts [32]. Osteoid is slowly calcified [32] and stiff substrate (150 kPa) was prepared to mimic bone matrix, which starts to calcify.

Type I collagen was coated on the substrate surface. Cells recognize and bind to type I collagen by integrin. Integrin is one of the adhesion proteins generally located on the cell membrane. Integrin binds to extracellular ligands, thereby integrating the cells with the microenvironment [23]. We observed the difference in integrin expression between pre-osteoblasts and fibroblasts. In pre-osteoblasts, the expression levels of α10- and α11-integrin were higher than fibroblasts, while the expression levels of αV- and β3-integrin were lower than fibroblasts (Figure 3b). Stan et al., reported that human osteoblast-like cells expressed higher level of α2- (mainly associated with type I collagen) compared to αV- (mainly associated with fibronectin) [40], while Thomas et al., reported that fibroblasts expressed higher level of αV- (mainly associated with fibronectin) compared to α2- (mainly associated with type I collagen) [41], which is consistent with our results. Integrins coordinate the assembly of the cytoskeleton and signaling complexes, and initiate distal functions [23]. For example, Shih et al., found that the increase in integrin quantity promotes the phosphorylation activation of focal adhesion kinase (FAK) and regulates cell differentiation [42]. We have demonstrated in our previous study that mesenchymal stem cells (MSCs) and fibroblasts express different levels of integrins’ subunits and this is attributed to different cell behaviors between MSCs and fibroblasts [43,44]. The expression levels of different integrins’ subunits in pre-osteoblasts and fibroblasts may also lead to different behavior on type I collagen-coated substrate.

We did not observe a significant spreading area and proliferation changes on Day 5 and Day 7 in pre-osteoblasts and fibroblasts on different substrates (Figure 4f,h and Figure 5). Our previous work showed that the spreading area of fibroblasts on 90 kPa substrate is close to the 12.3 kPa substrate [44]. Conleth et al., reported that pre-osteoblasts exhibited similar spreading areas on 19.6 kPa, 38.4 kPa and 153 kPa substrates [45]. Moreover, Liu et al., found no statistical difference in the proliferation rate of periodontal ligament stem cells on 16, 54 and 135 kPa substrates [46]. These previous results are consistent with ours. In addition, we did notice stiffness-dependent focal adhesion formation. Pre-osteoblasts formed longer focal adhesions as substrate stiffness increased from 15 kPa to 150 kPa (Figure 4a,b). Fibroblasts increased focal adhesion length as substrate stiffness increased from 15 kPa to 35 kPa and decreased focal adhesion length as substrate stiffness increased from 35 kPa to 150 kPa (Figure 4c,d). The expression level of integrin pairing to Type I collagen between pre-osteoblasts and fibroblasts may contribute to different optimal stiffness to form longer focal adhesion. There is an optimal stiffness for cells to form longer focal adhesion and the optimal stiffness is influenced by the integrin–ligand space [47]. In our result, higher number of integrins associated with type I collagen may lead to form longer focal adhesion in pre-osteoblast.

Gene expression did vary with substrate stiffness in two cell types. Our results show that pre-osteoblasts exhibited increasing Runx2 expression level with increasing stiffness (Figure 6a) and this is similar to the previous work of Zhang et al. [20]. It indicated that substrate stiffness can promote osteoblast differentiation. Additionally, we observed that substrate stiffness did not enhance myofibroblast differentiation, as there was no statistical difference in Acta2 expression of fibroblasts from 15 kPa to 150 kPa (Figure 6d). A previous study suggested that the level of Acta2 expression increased when substrate stiffness increased from 0.2 kPa to 8 kPa and did not increase when substrate stiffness increased from 8 kPa to 64 kPa [48], which is consistent with our results. Moreover, fibroblasts exhibited decreasing Fn1 and Cola1 expression with increasing stiffness (Figure 6e,f). Kim et al., show that fibronectin and collagen mRNA levels in human gingival fibroblasts are regulated by FAK/JNK signaling, while Acta2 expression is not [49]. It illustrates that substrate stiffness may also regulate fibronectin and collagen synthesis but does not regulate the expression of Acta2 through the downstream signaling pathway. Additionally, pre-osteoblasts exhibited decreasing OPN expression with increasing stiffness (Figure 6c). The downregulation of Fn1, Cola1 and OPN may be due to soft substrate generally enhancing cell secretory activity in vitro compared to stiff substrate [50]. Altogether, the observation of gene expression is an indicator that the stiffness change of bone-regeneration tissue (from 15 kPa to 150 kPa) promotes osteogenesis and cannot result in fibrosis.

In the physiological healing process, fibroblasts mainly perform their functions within the soft bone matrix at early stage. In the late repair stage, chondrocytes and osteoblasts perform functions within stiff bone matrix [9]. Our results suggested that substrate stiffness may be an important factor in the regulation of fibroblasts and pre-osteoblast function. It illustrated that, when fibroblasts and osteoblasts synthesize ECM proteins and increase the stiffness of bone ECM, bone ECM also regulates the function of fibroblasts and pre-osteoblasts.

Substrate stiffness generally regulates cell behavior by influencing biochemical signals, and this process is known as mechanotransduction [24]. Substrate stiffness regulates the contraction force of cell loading on the cytoskeleton and nucleus [47,51,52]. The contraction force of cell, which is known as the cell traction force (CTF), affects signaling pathways and cell behavior through mechanotransduction [26]. We found that the magnitude of the CTF generated by pre-osteoblasts is higher than that of the fibroblast (Figure 7). This may be due to the higher expression level of integrins binding to type I collagen in pre-osteoblasts (Figure 3b). Alberto Elosegui et al., reported that cells overexpressing the β6 integrin subunit, which combines with αV to form αVβ6 integrin and pairs to fibronectin [23], exhibited higher magnitude of CTF on 14 kPa and 29 kPa substrates coated by fibronectin [27], which is similar to our results. In addition, our results indicated that the CTF of pre-osteoblasts increased when the substrate stiffness increased (Figure 7b). Others have reported a similar increase in the CTF in human breast myoepithelial cells [27], which is consistent with our results. However, the CTF of fibroblasts increased as substrate stiffness increased from 15 kPa to 35 kPa and decreased as substrate stiffness increased from 35 kPa to 150 kPa (Figure 7d), though there is no statistical difference. This may be caused by adhesion collapse of fibroblasts because insufficient integrin on the cell membrane does not support high traction force [47]. Roger Oria et al., examined the CTF in human breast myoepithelial cells on surfaces of 1.5–150 kPa stiffness and saw a decrease in CTF from 30 kPa to 150 kPa [47], which is similar to our result. The subsequent pathways affected by the cell traction force will be carried out in our future study.

There are many types of cells that participated in bone regeneration and cellular responses to substrate stiffness which are different in different tissues [9,50]. Therefore, in the process of bone tissue engineering (BTE) scaffold construction, the control of cellular behavior using a biomimetic scaffold is a challenge [53]. Through the present results, we noticed that substrate stiffness is important because of the different effects of substrate stiffness on pre-osteoblasts and fibroblasts. A 150 kPa substrate promotes osteogenic differentiation, while it does not trigger an over-activation fibrotic response mediated by fibroblasts. Thus, it is recommended to adjust the stiffness of materials to around 150 kPa in BTE scaffold construction.

## 5. Conclusions

In conclusion, our results show that substrate stiffness is a regulator of pre-osteoblasts and fibroblasts. The substrate mimicking the stiffness of the bone-regeneration microenvironment can promote osteogenic differentiation of pre-osteoblasts, while it has no effect on myofibroblast differentiation of fibroblasts. It is reasonable to use a 150 kPa substrate for BTE scaffold construction. In addition, we find that substrate mimicking can regulate the formation of focal adhesions and CTF in pre-osteoblasts and fibroblasts. Our results improve the knowledge of how fibroblasts and pre-osteoblasts respond to the process of stiffness increasing in bone ECM from granulation tissue changing to soft callus and hard callus. Understanding the effect of the mechanical property of bone extracellular matrix provides us fresh cues to construct tissue engineering materials to mimic physiological stiffness and control multiple types of cells.

## Figures and Tables

**Table 1:** Composition of Acrylamide/Bisacrylamide for Preparation of Polyacrylamide Substrate.

	Soft	Middle	Stiff
40% Acrylamide (mL)	1.25	1.88	3.00
2% Bisacrylamide (mL)	0.75	1.50	3.00
10% Ammonium persulfate (mL)	0.1	0.1	0.1
TEMED (mL)	0.1	0.1	0.1
ddH2O (mL)	7.8	6.42	3.8

**Table 2:** Primer sequences for RT-PCR.

Target Gene	Forward Primer (5′-3′)	Reverse Primer (3′-5′)
Gapdh	AAGGTCGGTGTGAACGGATTTGG	CGTTGAATTTGCCGTGAGTGGAG
Runx2	TTTAGGGCGCATTCCTCATC	TGTCCTTGTGGATTAAAAGGACTTG
ALP	CGGGACTGGTACTCGGATAA	ATTCCACGTCGGTTCTGTTC
OPN	ATCTCACCATTCGGATGAGTCT	TGTAGGGACGATTGGAGTGAAA
Acta2	ATGCCTCTGGACGTACAACTG	CACACCATCTCCAGAGTCCA
Fn1	TACCAAGGTCAATCCACACCCC	CAGATGGCAAAAGAAAGCAGAGG
Itga1	CGCTGTGAATCAGACGAGGT	CCCACAGGGCTCATTCTTGT
Itga2	TGTCTGGCGTATAATGTTGGC	TGCTGTACTGAATACCCAAACTG
Itga5	TGCAGTGGTTCGGAGCAAC	TTTTCTGTGCGCCAGCTATAC
Itga10	AGGCCGAATTTGGATACAGTG	GAGCAACGATAAACATCCCCTC
Itga11	TGCCCCAATGGAAACCAATG	CATGCCAGTGGTGTAGTAGGA
ItgaV	CGGGTCCCGAGGGAAGTTA	TGGATGAGCATTCACATTTGAGA
Itgb1	ATGCCAAATCTTGCGGAGAAT	TTTGCTGCGATTGGTGACATT
Itgb3	GGCGTTGTTGTTGGAGAGTC	CTTCAGGTTACATCGGGGTGA

## Data Availability

The data used to support the findings of this study will be available from the corresponding author upon request.
